# Virus evolution and transmission in an ever more connected world

**DOI:** 10.1098/rspb.2014.2878

**Published:** 2015-12-22

**Authors:** Oliver G. Pybus, Andrew J. Tatem, Philippe Lemey

**Affiliations:** 1 Department of Zoology, University of Oxford, South Parks Road, Oxford OX1 3PS, UK; 2 Department of Geography and Environment, University of Southampton, Highfield, Southampton SO17 1BJ, UK; 3 Fogarty International Center, National Institutes of Health, Bethesda, MA, USA; 4 Flowminder Foundation, Stockholm, Sweden; 5 Department of Microbiology and Immunology, Rega Institute, KU Leuven—University of Leuven, Leuven, Belgium

**Keywords:** virus, epidemiology, geography, evolution, phylogenetics, transmission

## Abstract

The frequency and global impact of infectious disease outbreaks, particularly those caused by emerging viruses, demonstrate the need for a better understanding of how spatial ecology and pathogen evolution jointly shape epidemic dynamics. Advances in computational techniques and the increasing availability of genetic and geospatial data are helping to address this problem, particularly when both information sources are combined. Here, we review research at the intersection of evolutionary biology, human geography and epidemiology that is working towards an integrated view of spatial incidence, host mobility and viral genetic diversity. We first discuss how empirical studies have combined viral spatial and genetic data, focusing particularly on the contribution of evolutionary analyses to epidemiology and disease control. Second, we explore the interplay between virus evolution and global dispersal in more depth for two pathogens: human influenza A virus and chikungunya virus. We discuss the opportunities for future research arising from new analyses of human transportation and trade networks, as well as the associated challenges in accessing and sharing relevant spatial and genetic data.

## 1. Introduction

The consequences of international trade and travel for the dynamics of infectious disease are appreciated by researchers and the general public alike. In a highly mobile world, with over half a million travellers in the air at any one moment, viruses have more opportunities than ever before to disseminate globally. Growth in the reach, volume and speed of human mobility over the past century has connected pathogens with new and growing host populations, and contributed to a boom in emerging and re-emerging epidemics [1,2].

The increasing connectivity of our world affects transmission in many ways. Greater mobility, through business travel, tourism and labour movement, leads to more pathogen introductions, while social and ecological changes in recipient locations may raise the likelihood that introductions will become entrenched rather than die out. The establishment of new travel routes between previously unconnected locations also contributes. For example, direct air travel between South America, Africa and southeast Asia now links tropical continental regions, where infectious disease burdens are higher and year-round transmission is more common. Further, the increasing volume of global trade through shipping and air freight can spread contaminated goods or introduce disease vectors such as mosquitoes to new locations through accidental carriage (e.g. [3]).

Despite the importance of geography for infectious disease epidemiology, the effects of global mobility upon the genetic diversity and molecular evolution of pathogens are under-appreciated and only beginning to be understood; indeed, a recent monograph on the spatial epidemiology of infectious disease makes no reference to pathogen genetic variation [4]. Patterns of host mobility may be particularly important for RNA viruses, the infections on which we focus here. Because many viruses do not survive for long outside the environment of their host, close proximity of hosts (or of hosts and vectors) is often necessary for transmission. Further, because rates of RNA virus mutation and evolution are high, their genomes can accrue genetic differences while being spatially disseminated during an individual outbreak. The evolutionary and spatial dynamics of these pathogens are therefore linked and reciprocally influence each other [5,6]. This fundamental principle has several important consequences. First, genetic differences among viruses sampled from diverse locations will contain information about the spatial processes that gave rise to the virus's geographical distribution. The abundance of viral gene sequences and advances in analytical methods have increased our ability to infer these processes and track viral spread [6]. Second, rapidly evolving viruses are capable of adapting swiftly to the novel environments they encounter as they spread geographically [7], with the potential to alter, for example, vector specificity or sensitivity to drugs or immune responses. Third, spatial sampling provides a common frame of reference whereby virus evolution and migration can be integrated with epidemiological data, or with environmental measurements such as humidity or land use. Integration of geographical data with genetic analysis promises to provide a fuller understanding of the origins, dispersal and dynamics of evolving pathogens [8].

In this article, we explore each of these themes. We first review how spatial and genetic data are combined in empirical studies of viral transmission. Later we discuss in depth two human pathogens, influenza A virus (IAV) and chikungunya virus (CHIKV), whose global dynamics depend critically on the reciprocal interplay between virus evolution, spatial ecology and host mobility.

## 2. Methods for combining viral spatial and genetic data

Since the contemporary spatial distribution of a fast-evolving virus is the result of interacting ecological and evolutionary processes, consideration of spatial incidence or genetic data in isolation may provide only partial insight into the underlying transmission dynamics [5,9]. Consequently, there is considerable interest in the development new analytical methods, formal and informal, that combine both sources of information.

Several trends in technology and data availability over the last decade have spurred innovation in this area. The advent of cheap, mobile global positioning systems and their widespread adoption in disease surveys has revolutionized the geospatial recording and analysis of infectious disease incidence and prevalence, especially when combined with geographical information systems (GIS) and pervasive electronic communication [10]. Further, a wide range of data (e.g. high-resolution satellite images) that depict environmental, infrastructural and socio-economic variables that may determine disease dynamics are now available. Statistical models have been developed to exploit the relationships between these variables and geo-located disease data, and to predict the spatial distribution of infectious diseases (e.g. [11,12]). Of particular relevance to viruses are new insights into human mobility, generated by the analysis of datasets that describe global air travel passenger numbers [13,14], movements of marked banknotes [15] and anonymized mobile phone call records [16]. The latter have the potential to untangle human mobility in unprecedented detail and have been used to track population mobility following disasters [17], predict infectious disease dynamics [18] and plan disease elimination strategies [19]. At the same time as this progress in disease geography, viral gene sequences have greatly increased in abundance and length, in large part due to the adoption by virologists of next-generation sequencing technologies [20] that typically generate whole viral genomes rather than sub-genomic sequence fragments. Reported pathogen genomes are now more likely to be annotated with locations and dates of sampling, and for the most intensively studied species, such as HIV-1 and influenza, more than 100 000 virus sequences are publicly available.

The term ‘phylogeography’ is commonly applied to studies that use evolutionary trees to combine genetic data with spatial information [21]. Other statistical methods for examining the spatial distribution of genetic variation do not explicitly use phylogenies and are better described as ‘spatial genetics' (reviewed in [22]), while some genealogical approaches to population genetics combine aspects of both approaches (e.g. [23]). Phylogenetic methods are commonly applied to emerging viral epidemics, partly because the rapid evolution of such pathogens can create sufficient genetic variation for analysis at the level of individual infections, even during the early stages of an outbreak, and also because alternative population genetic approaches typically assume that mutation is negligible or that the processes of genetic drift and migration are in equilibrium [24]. The latter were developed with animal or plant populations in mind and may not adequately represent the idiosyncratic and dynamic dispersal histories that characterize ecological invasions [25,26]. Further, a single evolutionary tree (with associated estimation uncertainty) is often sufficient to represent the shared ancestry of all sites in a RNA virus sequence, owing to the absence or low rate of recombination within them.

Methods that attempt to combine viral genetic and geographical information will be worthwhile only if the spatial epidemiology of the pathogen population is recorded in its genome sequences. The degree to which that occurs for the pathogen in question will depend on its relative rates of spatial movement and molecular evolution. A pair of typical RNA virus genomes will diverge genetically from each other on average at a rate of 1–20 nucleotide changes per year (assuming 10^−3^–10^−4^ substitutions per site per year and a genome 10 000–20 000 nucleotides long [27]). Hence, to a very rough approximation, analyses of viral genomes are unlikely to contain a reliable record of spatial epidemiological trends that occur on time scales faster than a fortnight. It is therefore unsurprising that many studies focus on global or regional patterns, observed over a time scale of several years or decades. Transmission dynamics over short time scales can sometimes be partially resolved by augmenting viral gene sequences with epidemiological incidence data (e.g. [28,29]). It is also possible for virus sequences, particularly those limited to the antigenic regions of capsid or envelope proteins, to evolve too quickly relative to the rate of geographical spread, in which case phylogeographic information is lost due to the mutational ‘saturation’ of informative sites in viral genes (e.g. [30]). In general, the rate of pathogen molecular evolution will determine the time scale of the spatial processes that can be reliably inferred; for example, movement of influenza virus can, under the best circumstances, be pinpointed from whole-genome sequences to within a few weeks, whereas geographical trends in the diversity of much slower-evolving *Helicobacter pylori* genes reveal the global spread of the bacterium over more than 50 000 years [31].

Several of the most popular phylogeographic methods for reconstructing epidemic spatial spread from genetic data (e.g. [26,32–34]) treat the location information assigned to each sequence as a discrete or continuous trait, and represent movement as change in that trait along sampled lineages, using stochastic models that are uncoupled from the processes of molecular evolution. The focus is therefore on the locations and ages of sampled lineages rather than on underlying population genetic processes of selection, genetic drift and migration, an approach that may be viewed philosophically as either a strength or a weakness, depending on one's perspective and interests [21,35]. This ‘trait evolution’ approach to phylogeography facilitates the inference of the locations of common ancestors in an epidemic and can be practically applied to rapidly evolving pathogens with complex spatial dynamics [34]. Further, the inferred changes in location on a phylogeny are statistically independent observations, whereas the sample locations themselves are correlated due to their shared ancestry.

However, it is not always fully recognized that the estimated locations of ancestors can be highly uncertain, particularly those that are only distantly related to the sampled cases. Consequently, viral phylogeography is far more informative when applied to datasets that contain genetic sequences sampled sequentially through time, and which include genomes situated close to the root of the sample phylogeny. A second under-appreciated aspect of phylogeographic analysis is the importance of sample composition [36]. Although a highly detailed spatio-temporal record may not be required to address every important question about pathogen spread, the accuracy with which gene sequences can capture key patterns will depend on the representativeness of sampling. If samples from key locations or regions are absent or rare then virus movement will be underestimated and the inferred locations of ancestors may be biased towards locations that are over-represented in the sample. As a result, phylogeographic results should be interpreted carefully, combined with other sources of epidemiological information and statistically validated whenever possible.

## 3. Integration of viral spatial and genetic data in practice

The simplest way to combine viral spatial and genetic data is through the mapping of infections attributable to different viral strains. This creates a link to genetic variation because RNA viruses are classified into genotypes and subtypes using analysis of their gene sequences. In recent years, the global geographical distribution of strains of HIV-1 [37], dengue virus [11] and hepatitis B and C viruses [38,39] have been characterized in this way. Despite being primarily descriptive, such studies can be useful in public health planning. For example, severe disease following dengue virus infection is more common in regions where two or more viral serotypes co-circulate, and the success rate of drug treatment for hepatitis C virus infection varies significantly among viral genotypes.

Evolutionary analysis of viral genes can be used to validate the putative source of an emerging viral outbreak that has been identified through epidemiological surveillance and contact tracing. For example, the proposed index case of the 2007 outbreak of CHIKV in northeast Italy had hosted a relative from Kerala, India (where the virus was epidemic), and phylogenetic analysis of virus E1 gene sequences from the Italian outbreak showed it to be very closely related to strains previously isolated in India [40]. Independent testing of an outbreak's source using viral genetics is especially valuable when surveillance data is uncertain or absent, and may become commonplace as viral genome sequencing becomes routine in clinical diagnosis. It is therefore important that public health agencies recruit and retain expertise in the evolutionary analysis of pathogen genetic variation.

In addition to its confirmatory role, analysis of virus genomes can answer questions of relevance to infectious disease control that cannot be addressed using incidence reports alone. For example, viral phylogenies can indicate if an outbreak in a specific region is the result of a single introduction followed by onward transmission within the host population of that region, or is composed of multiple independent chains of transmission, each initiated by a separate introduction from elsewhere or from a zoonotic reservoir species. For example, analysis of viral genomes from the Ebola epidemic in west Africa that began in Guinea in early 2014 indicated that it developed from a single introduction from the virus's reservoir in central Africa, and that the epidemic in Sierra Leone arose from the transmission of two distinct viral lineages from Guinea [41]. By contrast, phylogenetic investigation of the HIV-1 subtype B epidemic in the UK showed that it comprised hundreds of independent viral introductions from other countries, at least six of which established large and persistent chains of transmission in the UK [42]. Epidemiological differences among observed transmission chains can help to focus epidemic control efforts more efficiently on specific populations or risk groups. Further epidemiological insights can be obtained by using evolutionary ‘molecular clock’ models, which place viral phylogenies on a real time scale of months and years [8], and enable estimation of the age of the most recent common ancestor (MRCA) of transmission chains in different locations. It is not always appreciated that the MRCA of an outbreak does not necessarily represent the same infected individual as the index case; the former can be more recent (but never older) than the latter. Despite this condition, estimated MRCA ages are sometimes weeks to years earlier than reported dates of virus discovery. Thus, this difference indicates a ‘time lag’ of epidemiological surveillance, the duration of which might be used to evaluate the efficiency and timeliness of systems of pathogen detection and notification.

If transmission is predominantly local and movement unimpeded by geographical barriers then the genetic and geographical distances among sampled infections are expected to be positively correlated. This principle, known as isolation by distance [24], forms a simple yet direct link between genetic and spatial information, and represents an important null hypothesis in spatial genetics. Strong correlations may be observed for viruses that disperse gradually, such as rice yellow mottle virus during its spread across sub-Saharan Africa [43]. However, patterns of isolation by distance can be swiftly lost if landscape features affect the dynamics of spread. A study of Zaire ebolavirus in central Africa suggested that the epizootic underwent an abrupt change in direction at a major biogeographic river barrier [44]. Rerouting geographical distances between virus sequences through this ‘pivot point’ led to much stronger correlations of genetic and geographical distances than when straight line distances were used [44]. Evidence for isolation by distance may be also eroded by high rates of host movement (a topic discussed later in the context of influenza viruses). The Ebola study, and others (e.g. [45]), illustrate the importance of using the locations of ancestral infections when reconstructing the geographical distance travelled by the chain of transmission that connects two sampled cases, especially when dissemination is not uniform in space. As discussed above, ancestral locations are typically inferred using one of the ‘trait evolution’ phylogeography methods.

Highly heterogeneous dispersal may be a common feature of all ecological invasions [46]. This variation has been accommodated in phylogeographic analysis using ‘relaxed random walk’ models that allow dispersal rates to vary significantly among phylogeny branches [34]. Application of this approach to the West Nile virus invasion of North America that began in New York in 1999 revealed that the epidemic was driven by a heterogeneous mix of local transmission and rare, long-range viral movements that probably represent seasonal migration of birds, the natural hosts of the virus [47]. An important consequence of such approaches is that each phylogeny branch becomes an independent observation of viral translocation, conditional on the data. This enables spatial epidemiological parameters, such as the epidemic diffusion coefficient and wavefront velocity, to be readily estimated from viral genome sequences alone [47].

A key goal of viral phylogeography is to help predict future pathogen spread by indicating those social or environmental factors that are associated with virus movement. This is often achieved by qualitative comparison of virus genetic diversity or dispersal history with geographical data. For example, the early spread of HIV-1 in east Africa was explored by combining phylogenetic analyses with regional data on road network architecture and population density, obtained using GIS techniques [48]. More recent phylogeographic studies have formalized this approach by parametrizing location exchange rates as a function of different potential causal factors, so that the effects of these drivers of spatial spread can be quantified and tested using genetic data [49]. Crucially, this enables virus genomes and host mobility data to be combined in a single statistical framework. Retrospective application of this technique to the 2009 influenza A pandemic demonstrated that combining both data sources predicted the global dissemination of the pandemic better than either alone [49].

## 4. Case study: influenza virus

**Figure 1.**
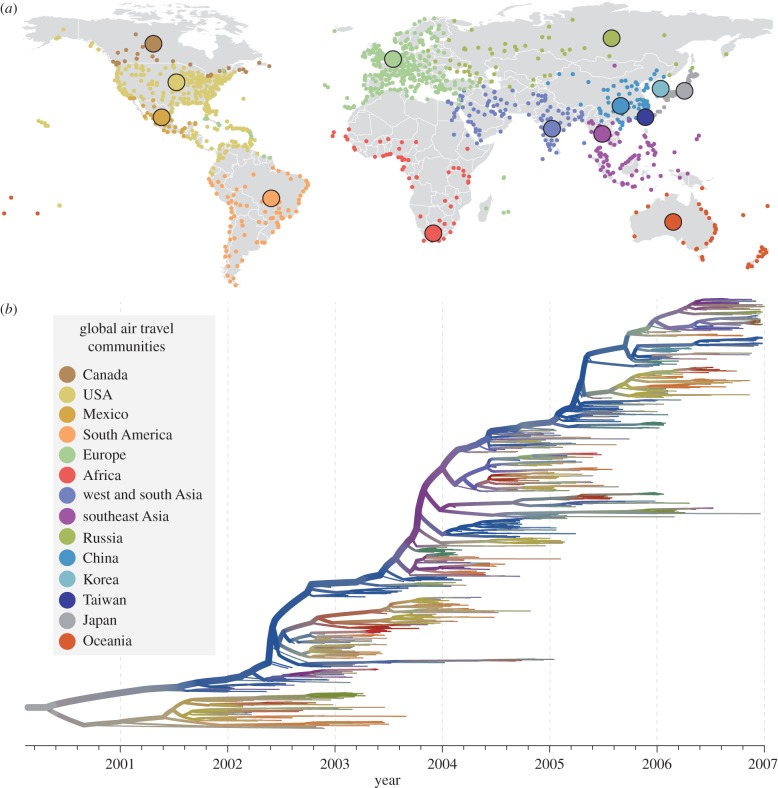
(*a*) The modular structure of global air travel. Airports (small dots) can be grouped into 14 communities (colours; inset) such that there is high connectivity within communities but low connectivity among them (hence French Guiana belongs to the European, not South American, community). Larger circles indicate the approximate geographical centre of each community. (*b*) A phylogeny of the H3N2 subtype of human IAV, estimated from more than 1000 virus haemagglutinin gene sequences that were sampled worldwide between 2002 and 2007. A molecular clock model was used, hence phylogeny branches represent time (time scale shown below the tree). The thickness of each branch is proportional to its number of descendent tips (up to a maximum thickness) and indicates lineage persistence. Each phylogeny branch is coloured according to its most probable location, which was inferred using a phylogeographic model that takes into account the global air travel network. The thicker, uppermost lineage represents the most persistent lineage of H3N2 influenza, which, for most years, is estimated to be located in southeast or east Asia. Figure adapted from Lemey *et al.* [49].

In addition to generating information essential for vaccine design, the global surveillance of influenza viruses has resulted in an unparalleled collection of virus genome sequences sampled through space and time, providing an opportunity to explore the processes that underpin the global dynamics of this important pathogen [50]. Although human influenza is primarily transmitted in household and community settings, epidemics of IAVs in temperate climates are seasonal and experience strong genetic bottlenecks, implying that transmission in these locations is typically not sustained and that epidemics are re-established by the importation of viral lineages from populations in which transmission is more persistent [51–53]. This so-called ‘source–sink’ model of global IAV circulation has been investigated in detail for the H3N2 subtype of IAV (figure 1), a dominant strain of human influenza since its emergence in 1968. Various studies have used phylogeographic and population genetic methods to infer the location through time of the ‘source’ population of H3N2 influenza, and most conclude that it resides primarily in east or southeast Asia [49,52,54] (figure 1). However, temperate regions, particularly the USA, may also contribute as a source [55], and there is evidence for viral gene flow into Asia from elsewhere [56], suggesting that the migration dynamics of H3N2 influenza are more complex than those represented by a simple source–sink model. Differences among these studies may however be attributable to variation in analysis methodology and sequence sampling strategy; seasonal fluctuations in sampling and the comparative under-sampling of IAV from south Asia, Africa and Latin America means that conclusions should be interpreted carefully [36]. Nevertheless, all analyses implicate global mobility as a driver of worldwide human influenza virus dispersal (figure 1); air passenger flux is a considerably better predictor of the movement of IAV lineages among locations than geographical distance [13,49]. Thus, the spatial genetics of human influenza, and possibly of other pathogens, may be better characterized by ‘proximity by mobility’ than by the traditional notion of ‘isolation by distance’.

The emergence of pandemic H1N1 (pH1N1) influenza in 2009 was the first influenza pandemic in the post-genomic era. Genetic analysis of the pandemic in its early stages was aided by pre-planned and intensive virus sequencing in some countries, and by the immediate and open sharing of the resulting data through online databases. Consequently, the molecular epidemiology of the virus could be tracked in ‘real time’ as the epidemic unfolded [57,58]. This included phylogeographic analyses that studied the global dispersal of the virus during its establishment phase [59,60], which followed patterns of international air travel [13,61]. The intensive sampling of virus sequences during the pandemic enabled the molecular epidemiology of IAV to be scrutinized at such a high resolution that the importation, extinction and persistence of individual transmission chains in specific locations could be observed (e.g. [62–64]). Comparisons among countries of the dynamics of transmission chains may provide useful insights. For example, only two of many pH1N1 lineages that were introduced to the UK at the start of the pandemic were detected there six months later [64], while a pair of pH1N1 transmission chains appear to have persisted in west Africa for almost 2 years [65]. The latter observation seems to be at odds with the extensive spatial mixing of IAV imposed by air travel, but west Africa is connected comparatively weakly within the global air transportation network [66] and influenza persistence might be facilitated there by climatic variability that can generate temporal overlap among epidemics in neighbouring regions [65], as has been previously suggested for IAV persistence in southeast Asia [52].

Local persistence of transmission chains also raises questions about the mobility processes that drive IAV spread at sub-national scales. Mathematical analyses of mortality and physician visit statistics have suggested different drivers for the spread of seasonal [67,68] and pandemic [69] influenza across the continental US. These studies variably emphasized the relative importance of workplace commuting [68], domestic airline travel [67] and school opening dates [69]. As an independent source of information about transmission, viral genetic data could help to resolve this problem. However, it is possible that sub-genomic influenza haemagglutinin gene sequences do not contain sufficient information to answer fine-scaled questions about viral dispersal over very short time scales. Instead, complete viral genome sequences will probably be needed to achieve the phylogeographic resolution required.

The spatial dynamics of influenza are also critical in assessing the evolution of anti-viral drug resistance. The global cycling of IAV lineages and low levels of local persistence mean that resistance mutations can spread worldwide, and can quickly erode any association at the local level between rates of drug usage and viral resistance. Recent examples of anti-viral drug resistance evolution include the rapid spread in oseltamivir resistance in seasonal H1N1 influenza from 2007 to 2009 and the global rise of adamantane-resistant H3N2 influenza during 2003–2006. An investigation of the former that used a stochastic model of international air travel concluded that the oseltamivir-resistant strain rose to global dominance because it exhibited a transmission advantage in untreated hosts, probably conferred by genetic hitchhiking [70]. Phylogeographic analysis of adamantane resistance in A/H3N2 IAV has shown that resistance evolved independently 11 times over 10 years [71], yet most of the resistant viruses found were descended from a single resistant lineage that was first detected in southeast Asia in 2003, before later spreading worldwide, consistent with the above-mentioned ‘source–sink’ model of global IAV circulation.

## 5. Case study: chikungunya virus

CHIKV is a mosquito-borne alphavirus that, while rarely fatal, causes a debilitating fever and sometimes persistent arthralgia, so is of some public health concern. In the 50 years that followed the virus's discovery in 1952 in Tanzania, sporadic outbreaks were reported in central, west and east Africa, and in south and southeast Asia [72]. However, the last decade has seen an increase in the geographical range of CHIKV. Starting from east Africa in 2004, CHIKV epidemics were reported increasingly eastwards, first on Indian Ocean islands (Comoros, Reunion, Seychelles and Mauritius) in 2005–2006, then in India and Sri Lanka in 2006–2007 [73]. Numerous countries in temperate regions have reported imported cases, one of which, in Italy, caused an autochthonous epidemic [40]. However, it is only within the last 18 months that CHIKV has finally become established in the New World. More than 750 000 suspected cases in the Americas have been reported since the detection of CHIKV on the Caribbean island of Saint Martin in December 2013, and several mathematical models that use data on human mobility and vector distributions have already been developed to predict further spread of the virus in the Americas (e.g. [74]).

**Figure 2.**
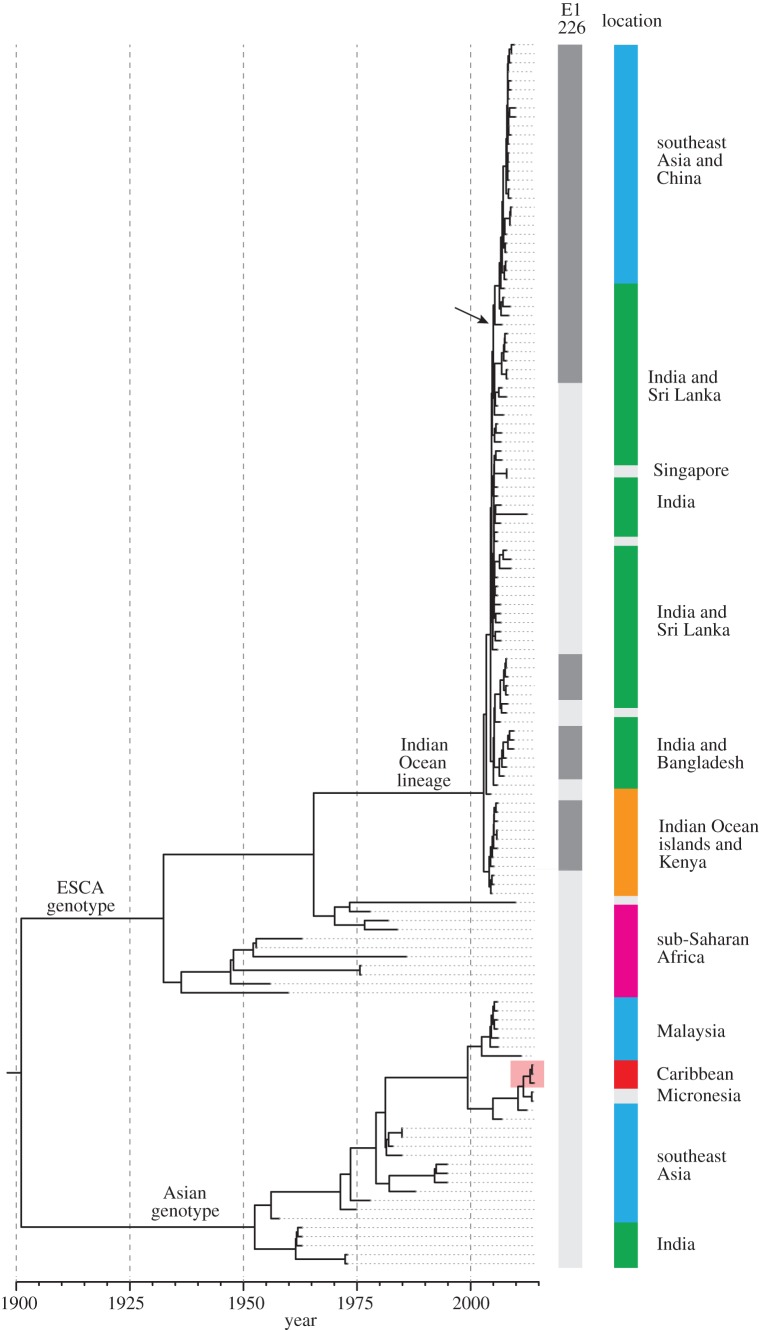
The evolution and global spread of CHIKV. On the left is a phylogeny of CHIKV, estimated from whole genomes of viruses sampled from the 1960s to the present day. Major CHIKV lineages are denoted (the west Africa genotype is not shown). The first vertical bar on the right indicates the amino acid present at position 226 in the CHIKV E1 protein (dark grey, valine; light grey, alanine). A change to valine at this site confers increased transmissibility of the virus in *Aedes albopictus* mosquitoes (see main text). The second vertical bar indicates the geographical location of the viruses (green, south Asia; blue, southeast Asia or China; orange, east Africa or Indian Ocean islands; purple, sub-Saharan Africa; red, Americas; grey, other locations). For returning travellers, the location of infection (not the location of detection) is shown. An arrow indicates the strain that caused an outbreak in Italy in 2007 (see main text). A red box indicates the lineage responsible for the recent emergence of CHIKV in the Americas.

The worldwide expansion of CHIKV has left a clear footprint in the genomic diversity of the virus, despite the fact that its rate of molecular evolution is somewhat slower than that of viruses like influenza and HIV [75]. Phylogeographic analysis of CHIKV genomes (figure 2) shows that two virus lineages (the ‘Asian genotype’ and the ‘Indian Ocean lineage’) were responsible for the recent expansion of its geographical range. The Asian genotype, first detected in India in the 1960s, is the strain that has recently emerged in the Caribbean and appears to have reached there via southeast Asia and Micronesia. By contrast, the Indian Ocean lineage was responsible for the significant epidemics in south Asia from 2005 onwards (figure 2) [75].

Multiple genetic and ecological factors are thought to have contributed to the global emergence of CHIKV. The two mosquito species principally responsible for human CHIKV transmission are *Aedes aegypti* and *Ae. albopictus.* The collapse of *Ae. aegypti* elimination efforts in the Americas [76] and growing urbanization in the tropics and sub-tropics has provided suitable habitats for this primary vector. Additionally, the globalization of trade in used tyres during the 1980s and 1990s enabled the secondary vector *Ae. albopictus* to expand its range from southeast Asia to large parts of the rest of the world [3]. Further, greater human travel between Africa, Asia and the Americas has increased interchange between locations where *Aedes* mosquitoes are prevalent, including at times of the year when the vectors are highly active in both places [73,77].

In addition to these ecological factors, there is strong evidence that, as the geographical range of CHIKV expanded, the virus evolved and adapted to local variation in the distribution of vector species. Specifically, a single amino acid change (A226V) in the viral E1 protein has been shown to increase transmission and infectivity in *Ae. albopictus* mosquitoes [78]. This mutation arose multiple times within the Indian Ocean lineage, usually in locations where *Ae. albopictus* was the sole or dominant vector species [79], and thus represents a remarkable example of convergent molecular evolution (figure 2). Fortunately, the Asian lineage that has recently emerged in the Americas has, to date, shown no propensity to evolve mutations that elevate transmissibility in *Ae. albopictus* mosquitoes.

## 6. Discussion

Our understanding and evaluation of the risks of infectious disease spread are being refined by access to growing geographically referenced databases of disease prevalence, detailed satellite-based imagery and unprecedented information about patterns of human mobility. Successful integration of these sources of information with viral genetic data will be technically and intellectually challenging, yet holds great promise for our response to emerging viruses.

Recent modelling work indicates that pathogen diffusion becomes highly regular when measured against a so-called ‘effective distance’ along the relevant mobility or transport network [13]. Conceptually, this requires translating from variable rates of spread through a space defined by geographical distances, to regular diffusion through a space defined by effective distances. The former process is already accommodated by phylogeographic analysis [34] so implementation of the latter should be possible. This work suggests that empirically derived networks of contacts among hosts may constitute a third common frame of reference by which genetic and epidemiological data can be unified, supplementing the temporal and spatial dimensions that are currently used [8]. In future, the concept of effective distances could be extended to epizootic or vector-borne pathogens, for which landscape heterogeneity is more important than human contact networks. Previous work has already shown the possibility of defining ‘climatic distances' that account for differences among locations and seasons in their suitability for vector-borne disease transmission [3]. Integrating genetic data in this context will require a melding of phylogeographic and GIS techniques [80,81] in order to detect more subtle deviations from distance-dependent movement than those imposed by human transportation networks.

A significant obstacle to further progress is the availability and expense of some of the most powerful and relevant datasets. For air travel, origin–destination data derived from air ticket sales are available, but are highly expensive for research purposes, and their use may require legal and confidentiality agreements, resulting in a reliance on modelled datasets [14]. Moreover, detailed data on human mobility derived from mobile phone call records often prompt privacy and commercial concerns. Although virus genetic data are usually deposited in publicly accessible databases such as GenBank upon publication of the paper that report them, the delay between sequence generation and publication may prevent the opportunity to undertake real-time molecular epidemiology during an outbreak. Further, genetic data obtained by surveillance efforts may be reported without essential epidemiological information, such as the date and location of sampling, or may never be published at all, for reasons of commerce, politics or privacy. The success of GISAID (http://gisaid.org) and other initiatives in facilitating the timely sharing of influenza virus genomes during the 2009 H1N1 pandemic has unfortunately not been repeated in subsequent outbreaks. We strongly support the recent call for an international and inter-disciplinary consensus towards the open sharing and release of pathogen genetic information during epidemics [82].

New outbreaks of infectious disease, especially those caused by viruses, are a common phenomenon in the twenty-first century, and future trends in global mobility and trade seem likely to maintain or even accelerate their rate of appearance. Techniques and data to describe, explain and predict such occurrences can help to measure and mitigate the risks from novel and re-emerging pathogens. Statistical and mathematical models that integrate spatially explicit data on pathogen evolution with information on human movement and environmental variability have much to contribute to epidemic management, as well as deepening our understanding of fundamental evolutionary and ecological processes.

## Note added in proof

Since this review was written, the Asian genotype of CHIKV has spread from the Caribbean to Mexico, Brazil and Columbia, and local transmission has been observed in mainland France and Florida, USA. A second CHIKV genotype (ESCA) appears to have been introduced to Brazil from central Africa [83]. In addition, two recent studies have provided further insights into the interplay between human mobility and IAV evolution and transmission. Bozick & Real [84] showed that interstate commuter networks in the USA match the spatial genetic variation of IAV subtype H1N1. Bedford *et al*. [85] reported that age-dependent differences in infection and air travel frequency can explain the distinct evolutionary behaviours of influenza A and B viruses.
